# Dental Composites with Calcium / Strontium Phosphates and Polylysine

**DOI:** 10.1371/journal.pone.0164653

**Published:** 2016-10-11

**Authors:** Piyaphong Panpisut, Saad Liaqat, Eleni Zacharaki, Wendy Xia, Haralampos Petridis, Anne Margaret Young

**Affiliations:** 1 Department of Biomaterials and Tissue Engineering, UCL Eastman Dental Institute, London, United Kingdom; 2 Interdisciplinary Research Centre in Biomedical Materials (IRCBM), COMSATS, Lahore, Pakistan; 3 Department of Restorative Dentistry, Unit of Prosthodontics, UCL Eastman Dental Institute, London, United Kingdom; Institute of Materials Science, GERMANY

## Abstract

**Purpose:**

This study developed light cured dental composites with added monocalcium phosphate monohydrate (MCPM), tristrontium phosphate (TSrP) and antimicrobial polylysine (PLS). The aim was to produce composites that have enhanced water sorption induced expansion, can promote apatite precipitation and release polylysine.

**Materials and Methods:**

Experimental composite formulations consisted of light activated dimethacrylate monomers combined with 80 wt% powder. The powder phase contained a dental glass with and without PLS (2.5 wt%) and/or reactive phosphate fillers (15 wt% TSrP and 10 wt% MCPM). The commercial composite, Z250, was used as a control. Monomer conversion and calculated polymerization shrinkage were assessed using FTIR. Subsequent mass or volume changes in water versus simulated body fluid (SBF) were quantified using gravimetric studies. These were used, along with Raman and SEM, to assess apatite precipitation on the composite surface. PLS release was determined using UV spectroscopy. Furthermore, biaxial flexural strengths after 24 hours of SBF immersion were obtained.

**Results:**

Monomer conversion of the composites decreased upon the addition of phosphate fillers (from 76 to 64%) but was always higher than that of Z250 (54%). Phosphate addition increased water sorption induced expansion from 2 to 4% helping to balance the calculated polymerization shrinkage of ~ 3.4%. Phosphate addition promoted apatite precipitation from SBF. Polylysine increased the apatite layer thickness from ~ 10 to 20 μm after 4 weeks. The novel composites showed a burst release of PLS (3.7%) followed by diffusion-controlled release irrespective of phosphate addition. PLS and phosphates decreased strength from 154 MPa on average by 17% and 18%, respectively. All formulations, however, had greater strength than the ISO 4049 requirement of > 80 MPa.

**Conclusion:**

The addition of MCPM with TSrP promoted hygroscopic expansion, and apatite formation. These properties are expected to help compensate polymerization shrinkage and help remineralize demineralized dentin. Polylysine can be released from the composites at early time. This may kill residual bacteria.

## Introduction

Commonly used dental restorative materials include dental composite and amalgam. Following the 2013 Minamata Convention a multi-national phase-out of mercury-containing devices, including dental amalgam, has been agreed [1]. A number of clinical studies, however, have reported higher failure rates for dental composite restorations compared with dental amalgam [2–4]. The most frequent cause of composite failure has been recurrent (secondary) infection. This may result in continuing apatite dissolution beneath the restoration. It may occur if the cavity becomes unsealed due to polymerization shrinkage and there is bacterial ingress or residual infected dentin [5, 6]. A dental composite that swells to compensate shrinkage, promotes apatite precipitation from dentinal fluid and has antibacterial components could therefore be beneficial.

Water sorption-induced expansion and remineralizing action of dental composites can be encouraged through the incorporation of hygroscopic Mono Calcium Phosphate Monohydrate (MCPM) with Tri Calcium Phosphate (TCP) [7, 8]. These phosphates can additionally encourage the precipitation of apatite (brushite or hydroxyapatite) that may promote the remineralization of the demineralized dentin [9]. In other dental products, calcium has been replaced by strontium (Sr). Strontium ions can replace calcium in hydroxyapatite but in addition may provide antibacterial action and greater radiopacity [10, 11]. Calcium substitution by Sr in bioactive glasses for orthopedic applications has also been shown to stabilize hydroxyapatite precursor phases and crystalline growth [12]. Polylysine (ε-poly-L-lysine; PLS) is a small natural homopolymer, which has been approved by the FDA as a food preservative [13]. PLS has demonstrated a wide antimicrobial spectrum in addition to low toxicity to human cells [14].

The aim of this study was therefore to produce MCPM, tri strontium phosphate (TSrP) and PLS containing composites. Monomer conversion, calculated polymerization shrinkage, water sorption induced mass and volume change, material induced apatite precipitation and PLS release were assessed in addition to mechanical strength.

## Materials and Methods

### Composite paste preparation

Four experimental light activated dental composite formulations were prepared using a powder to liquid ratio of 4:1 (weight ratio). Chemicals used in this study are presented in Table 1. The monomer phase in all formulations was prepared by mixing UDMA and TEGDMA in 3:1 weight ratio with 1 wt% CQ, 1 wt% DMPT, and 5 wt% 4-META respectively. The powder phase of each formulation contained varying levels of Glass, MCPM, TSrP, and PLS (Table 2). A commercial dental composite (Z250 shade B3, 3M, USA) was used for comparison.

**Table 1:** Chemicals used in this study.

Abbreviation		Chemicals	Batch/lot number	Company
UDMA	Urethane dimethacrylate		90761	DMG, Hamburg, Germany
TEGDMA	Tri-ethylene glycol dimethacrylate,		88661	DMG, Hamburg, Germany
4-META	4-Methacryloyloxyethyl trimellitic anhydride		595697	Polysciences, PA, USA
CQ	Camphorquinone		90339	DMG, Hamburg, Germany
DMPT	N, N-dimethyl-p-toluidine		MKBR6240V	Sigma Aldrich, Gillingham,UK
Glass	Silanated bariumaluminosilicate glass, particle diameter ~ 7 μm		680326	DMG, Hamburg, Germany
MCPM	Monocalcium phosphate monohydrate, particle diameter ~ 53 μm		MCP-B26	Himed, Old Bethpage, NY,USA
TSrP	Tristrontium phosphate, particle diameter ~10 μm		EDI077976	Chemieliva, Chongqing,China
PLS	ε-Polylysine, particle diameter ~ 20–50 μm		128211-04-3	Handary, Brussel, Belgium

**Table 2:** Composition of powder phase of experimental composites.

Formulation (F)	Glass (wt%)	MCPM (wt%)	TSrP (wt%)	PLS (wt%)
1	72.5	10	15	2.5
2	75	10	15	0
3	97.5	0	0	2.5
4	100	0	0	0

The fillers and monomer of each formulation were weighed and mixed using a planar mixer (SpeedMixer, Synergy Devices Limited, UK) at 3500 rpm for 10 s followed by 2000 rpm for 2 min. The consistency of the mixed experimental composites was comparable to commercial packable composites.

### Monomer conversion and polymerization shrinkage

Monomer conversion was determined by using a Fourier transform infrared spectrometer (FTIR, Perkin-Elmer Series 2000, Beaconsfield, UK) equipped with a golden gate Attenuated Total Reflectance (ATR) accessory (3000 Series RS232, Specac Ltd., UK) at a controlled temperature of 25°C [8]. Uncured pastes (n = 5) were placed in a ring (1 mm depth and 10 mm diameter) on the ATR diamond, covered with acetate sheet, then light cured for 40 s from the top surface with a LED light curing unit (1,100–1,330 mW/cm^2^, Demi Plus, Kerr, USA). FTIR spectra of the bottom surfaces were recorded every 4 s for 20 min between 700–4000 cm^-1^, at a resolution of 4 cm^-1^. In this study conversion, *C*, was obtained using Eq 1.

$$C\;(\%)=\frac{100({\Delta A}_{0}-{\Delta A}_{t})}{{\Delta A}_{0}}$$ (1)

Where Δ*A*_0_ and Δ*A*_*t*_ were the absorbance of the C-O peak (1320 cm^-1^) above background level at 1335 cm^-1^ initially and after time *t*. Final peak height and degree of monomer conversion were calculated by linear extrapolation of the data versus inverse time to zero.

One mole of polymerizing C = C groups typically gives volumetric shrinkage of 23 cm^3^/mol [15]. Total percentage volume shrinkage (*φ*) (%) due to composite polymerization can therefore be estimated from monomer conversion using the following equation.

$$\varphi =23\;C\rho \;\sum_{i}\frac{n_{i}x_{i}}{W_{i}}$$ (2)

where *C*, monomer conversion (%); *ρ*, composite density (g/cm^3^); *n*_*i*_; the number of C = C bonds per molecule; *W*_*i*_, molecular weight (g/mol) of each monomer; *x*_*i*_, mass fraction of each monomer [8].

### Mass and volume changes

For all formulations, disc specimens were prepared by carefully packing the composite resin in a metal circlip (1 mm depth and 15 mm diameter, n = 3) and covering top and bottom with acetate sheet. Discs were light cured for 40 s on each side with the LED light curing unit. Specimens were then left for 24 hr at ambient temperature to allow completion of polymerization. Disc specimens were subsequently weighed and immersed in 10 ml of deionized water or simulated body fluid (SBF) prepared according to BS ISO 23317:2012 [16]. The tubes were incubated at 37°C for up to 9 weeks. At 1, 2, 4, 6, 12, 24, 48, 72, 96 hr and 1, 2, 3, 4, 6, 7, 8, 9 weeks the samples were removed and carefully blotted dry. Their mass and volume were subsequently measured using a four-figure balance (Ohaus PA214, Parsippany, NJ, USA) with density kit before replacement into the original storage solution. The percentage mass and volume change, ΔM and ΔV, were determined from Eqs 3 and 4 respectively [17].

$$\Delta M\;(\%)=\frac{100[M_{t}-M_{0}]}{M_{0}}$$ (3)

$$\Delta V(\%)=\frac{100[V_{t}-V_{0}]}{V_{0}}$$ (4)

where *M*_*t*_ and *V*_*t*_ are mass and volume at time *t* after immersion, whereas *M*_0_ and *V*_0_ is initial mass and volume.

### Apatite formation

The ability of the resin composites to promote apatite precipitation was assessed following the BS ISO 23317:2012. In order to characterize any changes in surface chemistry and apatite precipitation, Raman microscopy (Horiba Jobin Yvon, Paris, France) was employed. Briefly, disc specimens (1 mm deep and 15 mm diameter, n = 1) were immersed in SBF at 37°C for 24 hr, 1 week or 4 weeks. At each time point, specimens were removed, blot dried and secured on glass microscope slides. They were then excited at 633.8 nm by a He-Ne laser through a microscope objective (50x). Surface point spectra were obtained in the region of 800–1700 cm^-1^ using a confocal hole of 300 μm, giving an approximate spatial resolution of 5 μm in x, y and z directions. For single point spectra, the average of 20 spectra each of 10 s acquisition time was generated. To obtain Raman maps, point spectra were obtained every 4 μm over an area of 40x50 μm^2^. After baseline subtraction, spectra were normalized over the full spectral range and chemical maps generated using LabSpec 5 software. Using the full spectra of pure components, this program enables chemical maps to be generated even when, as with different phosphates, main peaks are partially overlapping. In the maps, different colors indicate which chemical component is the dominant phase at a given point on the material surface. The phosphate P-O stretch gives intense peaks for pure MCPM at 901, 912 and 1011 cm^-1^, brushite (dicalcium phosphate dihydrate) at 989 cm^-1^, TSrP at 948 cm^-1^ and apatite at 960 cm^-1^ [18]. The glass and polymer phase give peaks at 1370, 1400, and 1447 cm^-1^. Specimens used for Raman mapping were then coated with gold-palladium for imaging using a sputter coater (Polaron E5000, East Sussex, UK) for 90 s at 20 mA. Surface scanning was carried out using a scanning electron microscope (Phillip XL-30, Eindhoven, The Netherlands) operating with primary beam energy of 5 kV and a current of approximately 200 pA.

### Polylysine release

Disc specimens (1 mm x 15 mm, n = 3) were stored in 10 ml of deionized water and incubated at 37°C. At each time point (1, 6 and 24 hr, 1, 2, 3, 4, 5, 6, and 7 weeks), the storage solution was removed for analysis and replaced with a fresh solution. A Trypan Blue (TB, Sigma Aldrich, Gillingham, UK) assay was modified to enable analysis of the PLS concentration [19]. This method involved mixing 80 ppm of TB in 0.02 MES (4-Morpholineethanesulfonic acid, Sigma Aldrich, Gillingham, UK) / 0.03 NaCl (Sigma Aldrich, Gillingham, UK) with an equal volume of sample storage solution. The resultant mix was incubated at 37°C for 1 hr to enable a precipitation reaction between TB and the PLS. After allowing for cooling to room temperature the mixture was centrifuged at 13000 rpm for 20 min. The remaining supernatant was carefully pipetted and analyzed using an ultraviolet / visible (UV) spectrometer (Unicam UV 500, Thermospectronic, Cambridge, UK). Absorbance between 300 and 800 nm due to unreacted TB was recorded. To assess the concentration of PLS that had reacted with the TB, the above procedure was repeated with solutions of known polylysine concentration (1, 2, 4, 5, 6, 7, 8, 9, and 10 ppm in deionized water). This enabled generation of a calibration curve of absorbance at 580 nm versus PLS concentration. The cumulative amount of PLS release (%) at time *t* was the calculated by Eq 5.

$$\%\;PLS\;release=\frac{100[\sum_{0}^{t}R_{t}]}{w_{c}}$$ (5)

where *w*_*c*_ is the amount of PLS (g) incorporated in the specimen, *R*_*t*_ is the amount of PLS released into each storage solution (g) collected at time *t*.

### Biaxial flexural strength (BFS) and modulus of elasticity

Prior to testing, specimens, prepared as above but of 1 mm x 10 mm (n = 8), were immersed in 10 ml of SBF for 24 hr in an incubator at 37°C. A “Ball on ring” jig was used with a computer-controlled universal testing machine (Shimadzu AGSX, Kyoto, Japan) [20]. The specimens’ thickness was measured using a digital vernier caliper (Moore & Wright, West Yorkshire, UK) and placed on a knife-edge ring support of 8 mm diameter. The load was applied using a 4 mm diameter spherical ball indenter at 1 mm.min^-1^ crosshead speed. The failure stress was recorded in N and the biaxial flexural strength (S; Pa) was calculated using the following equation:

$$S=\frac{F}{l^{2}}\left\{(1+\nu )[0.485ln(\frac{e}{l})+0.52]+0.48\right\}$$ (6)

where *F* is the load at failure (N), *l* is the specimens thickness (m), *e* is the radius of circular support (m) and *v* is Poison ‘s ratio (0.3) [20]. Then, the force versus displacement graph was also used to calculate the biaxial modulus of elasticity using Eq 7.

$$E=(\frac{\Delta J}{\Delta W_{c}})\times (\frac{\beta_{c}a^{2}}{h^{3}})$$ (7)

Where *E* is elastic modulus of the specimen (Pa), $\frac{\Delta J}{\Delta W_{c}}$ = rate of change of load with regards to central deflection or gradient of force versus displacement curve (N/m), *β*_*c*_ is centre deflection function and center deflection junction (0.5024)[21], h is ratio of support radius to the radius of disc, and *v* is Poison’s ratio (0.3).

### Statistical analysis

All values and errors reported throughout this study were mean and 95% confidence intervals respectively. Raw data are provided in S1 File. Data were analyzed with SPSS version 22 for Mac (IBM, USA). Monomer conversion and BFS were analyzed with a Kruskal-Wallis test, and post hoc comparison was performed using Dunnett’s T3 test (*p* = 0.05). Calculated polymerization shrinkage and modulus of elasticity were analyzed using one-way analysis of variance (ANOVA) followed by Tukey test (*p* = 0.05). Line fitting for regression analysis was undertaken using the function LINEST in Microsoft Excel. In addition, a factorial analysis method was employed to assess the effects of variables and variable interactions. The formulations in this study are based upon a 2 variables and 2 levels factorial design. An appropriate factorial equation is then

$$lnP=<lnP>\pm a_{1}\pm a_{2}\pm a_{1,2}$$ (8)

a_1_ and a_2_ quantifies the effect of the phosphate fillers (PO) and PLS addition on the property value (P) of the experimental composites, a_1,2_ is an interaction effect of PO and PLS addition, and brackets indicate an average value of In P [22]. The percentage effect of the variables, Q, is then calculated using:

$$Q(\%)=100\left(1-\frac{G_{H}}{G_{0}}\right)=100(1-\exp\left(2a_{i}\right))$$ (9)

G_H_ and G_0_ are the geometric average property (e.g. monomer conversion, mass change, volume change, BFS and modulus) for the 2 samples with one of the additives versus the other 2 without respectively. The effect of polylysine was therefore obtained by comparing average results of samples F1 and F3 with the average for F2 and F4. Effect of phosphates is gained by dividing the geometric average of F1 and F2 by that for F3 and F4. Finally, the interaction effect is calculated from the average value of samples F1 and F4 divided by that of F2 and F3.

95% confidence interval (*CI*) error bars were calculated assuming *CI* = 2*SD*/*√n* where *SD* is standard deviation and *n* is sample number. The effect of an additive was considered significant if the magnitude of *a*_*i*_ was greater than both its calculated 95% CI and the interaction term.

## Results

### Monomer conversion and polymerization shrinkage

**Fig 1:**
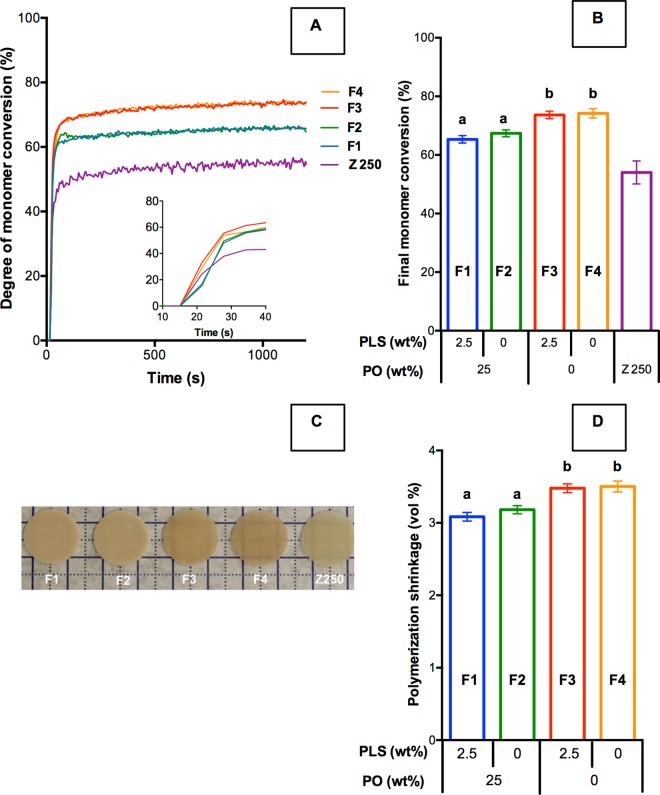
(A) Example polymerization profiles showing monomer conversion versus time of data acquisition (light exposure started at 10 s), (B) mean final monomer conversion, (C) composite discs after light curing showing different translucencies and (D) mean calculated polymerization shrinkage (same letters indicate no statistically significant differences (*p* < 0.05) and error bars are 95% confidence intervals (n = 5)).

All composites showed rapid monomer conversion up to 50% at the bottom of the specimens within 10 s after the start of light exposure (equal to 20 s after the start of data collection). From 20 s after the beginning of light exposure, reaction rate slowed substantially (Fig 1A). The final monomer conversions of experimental composites (64–76%) were significantly higher than Z250 (54 ± 3%) (Fig 1B). The final conversion of F1 (65 ± 1%) and F2 (67 ± 1%) were not significantly different from each other, but were significantly lower than F3 (74 ± 1%) and F4 (74 ± 1%). Translucency of the cured composites increased from F1 to F4. The translucency of F4 and Z250 materials was comparable (Fig 1C). The calculated shrinkage of F1, F2, F3, and F4 were 3.1 ± 0.1, 3.2 ± 0.1, 3.5 ± 0.1, and 3.5 ± 0.1 vol% respectively (Fig 1D). Shrinkage of Z250 could not be calculated because its exact composition was unknown. Factorial analysis showed the addition of phosphate filler decreased both of final monomer conversion and shrinkage by 10 ± 2% whilst the effect of PLS was negligible (2 ± 2%).

### Mass and volume change

**Fig 2:**
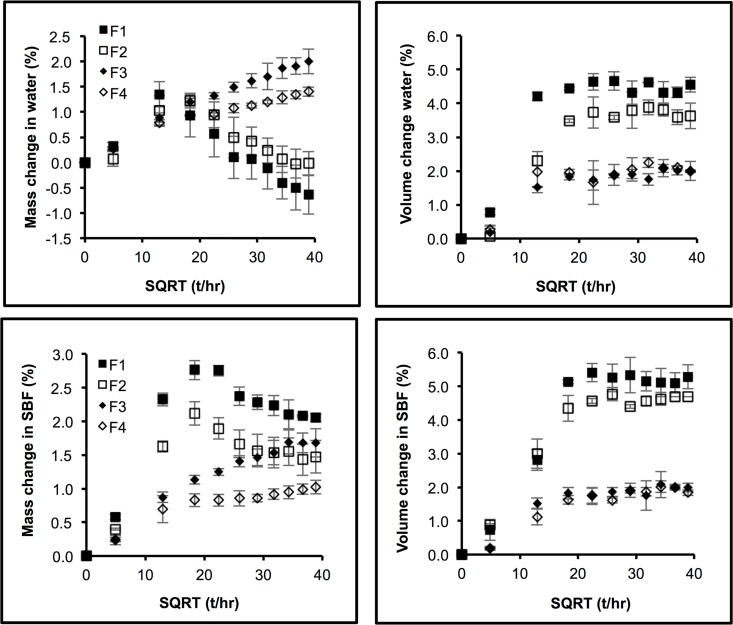
Mass and volume changes in SBF or deionized water as a function of square root of time (t/hr) of all formulations. Error bars are 95% CI (n = 3).

With F4, plots of mass change versus square root of time began to level off after 2 weeks (Fig 2). The final mass changes were 1.4 (± 0.1) and 1.0 (± 0.1) wt% in water and SBF respectively. Upon addition of PLS (F3) mass change continued for longer to final values of 2.0 (± 0.1) and 1.7 (± 0.2) wt% in water versus SBF respectively. Conversely, addition of phosphate filler (F1 or F2) caused a greater increase in early mass change but this peaked by 2 weeks. By 9 weeks, the mass changes had declined to values of 0.0 (± 0.2) and 1.5 (± 0.30) for F2 and -0.6 (± 0.4) and 2.0 (± 0.1) wt% for F1 in water versus SBF. The difference in mass change in water versus SBF was proportional to the square root of time (R^2^>0.95) with proportionality constants of 0.039 (± 0.002) and 0.075 (± 0.005) wt%/hr^-0.5^ for F2 and F1 respectively.

Conversely, volume change (Fig 2) reached plateau values in both fluids and all formulations at later times. The final values of volume change were 4.5 (± 0.2), 3.7 (± 0.1), 2.2 (± 0.2), 2.1 (± 0.1) vol% for F1, F2, F3 and F4 in water and 5.2 (± 0.1), 4.6 (± 0.1), 1.9 (± 0.1), 1.9 (± 0.1) vol% in SBF. Differences in final values in water versus SBF were therefore 0.7 (± 0.1), 0.9 (± 0.2), -0.4 (± 0.1), -0.1 (± 0.0) for F1, F2, F3, and F4 respectively.

### Apatite formation

**Fig 3:**
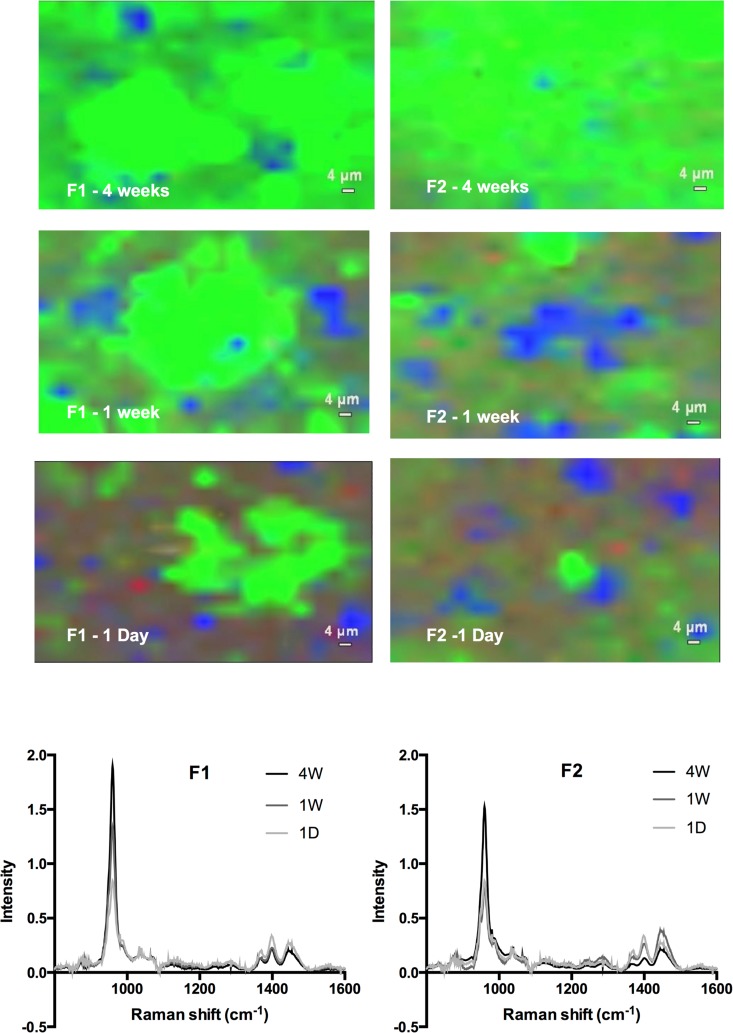
Average Raman spectra and surface Raman maps obtained from F1 and F2 at 1 day, 1 week, and 4 weeks. Blue and green areas represent the polymer plus glass regions versus an apatite coating respectively. Yellow and red areas represent regions of brushite and TSrP. No MCPM could be detected. At early times some blues areas are visible in the maps and the average spectra give strong polymer and glass peaks between 1300 and 1500 cm^-1^. A dominant apatite area and peak are observed for F1 at 1 week, but these can be seen for both formulations at 4 weeks.

After one day immersion in SBF, Raman spectra of F3 and F4 showed only peaks attributable to glass and polymers with no calcium phosphate peaks as was expected. At 1 day, peaks for glass, polymer and TSrP dominated the Raman spectra for F1 and F2 (Fig 3). The surface MCPM, however, had fully dissolved due to its high solubility. At this time, in Raman maps some areas of apatite were detected (green areas in maps in Fig 3) and in addition with F1 also a small amount of brushite (orange region). The average Raman apatite peak increased in intensity more rapidly with F1 than with F2. With F2, over 50% of the surface area examined was covered by a sufficiently thick layer (>1 μm) to fully mask the underlying composite by 4 weeks. Conversely, with F1 this occurred at 1 week.

**Fig 4:**
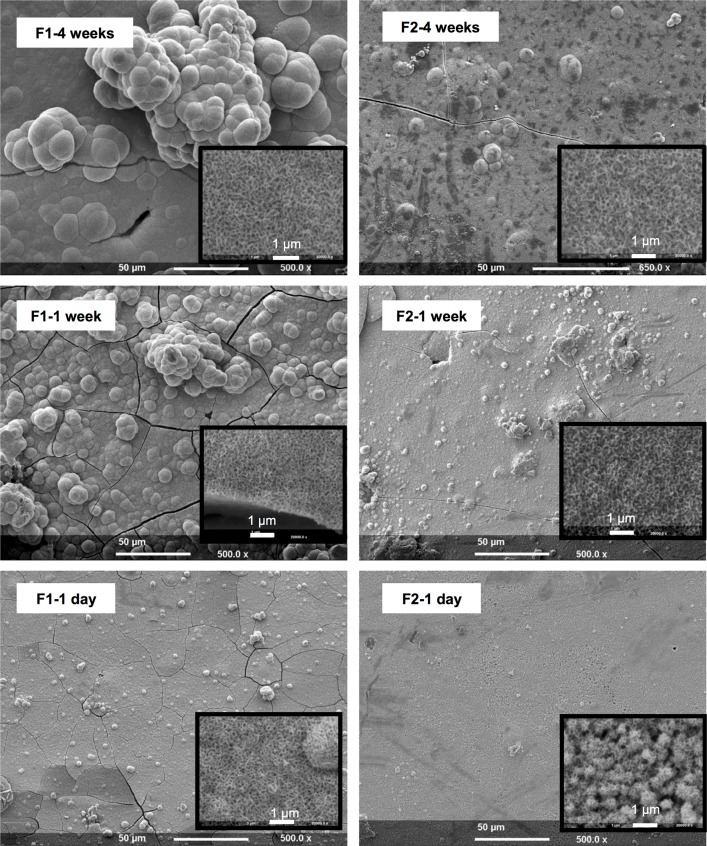
SEM images of F1 and F2 composite surfaces after submersion in SBF for 1 day, 1 week or 4 weeks. Cracks observed in the apatite layers were formed due to the coating process.

The SEM examination revealed a thin layer of apatite on the surfaces of F1 and F2 at all 3 time points of immersion (Fig 4), but no apatite was detected on the surfaces of the F3 and F4 groups. The apatite layers consisted of globules which at higher magnification indicated a porous structure. From the size of the glubules and dimensions of cracks in the apatite layers, the precipitate thicknesses were estimated to be approximately 1, 5 and 10 μm after 1 day, 1 week, and 4 weeks SBF immersion respectively for F2. With F1 the layer thickness was appoximately 2, 10, and 20 μm at these time periods.

### Polylysine release

**Fig 5:**
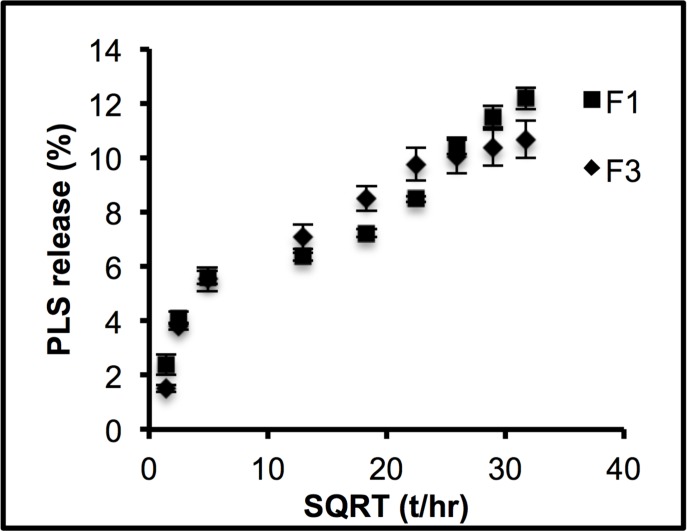
PLS release upon square root of time (hr) of formulations containing PLS (F1 and F3). Error bars are 95% CI (n = 3).

The composite formulations containing PLS (F1 and F3) exhibited a burst release of polylysine at early time points followed by release that was linear with the square root of time (Fig 5). Linear regression of data from 6 hr indicated a burst release of 3.4 ± 1.0% and 4.0 ± 0.8% and gradient of 0.26 ± 0.06%/hr^-0.5^ and 0.23 ± 0.04%/hr^-0.5^ for F1 and F3 respectively with R^2^ > 0.95 in both cases. Results for these two formulations were not significantly different.

### Biaxial flexural strength (BFS) and modulus of elasticity

**Fig 6:**
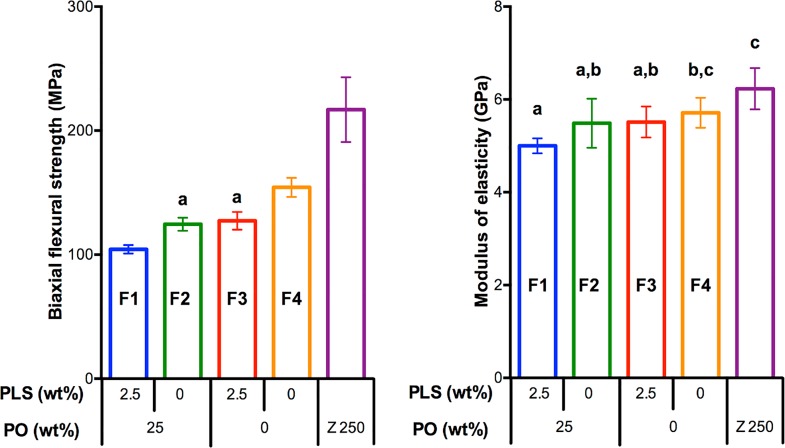
BFS and modulus of elasticity of each formulation. Same letters indicate no statistically significant differences (*p* < 0.05). Error bars are 95% CI (n = 8).

The highest and lowest BFS were obtained for Z250 (217 ± 22 MPa) and F1 (104 ± 3 MPa) respectively (Fig 6). F2 (125 ± 4 MPa) and F3 (127 ± 6 MPa) had comparable BFS but both were lower than F4 (154 ± 6 MPa). Z250 had the highest modulus (6.2 ± 0.4 GPa) but that of F4 (5.7 ± 0.3 GPa) was not significantly different. F1 (5.0 ± 0.1 GPa) showed the lowest modulus but this was not significantly different from that for F2 (5.5 ± 0.4 GPa) and F3 (5.5 ± 0.3 GPa). Factorial analysis indicated that adding PO and PLS decreased strength on average by 18 (± 3) % and 17 (± 3) % respectively with no variable interaction effect (1 ± 6%). In contrast, these additives had no significant effect on modulus of the experimental composites.

## Discussion

The objective of this study was to assess the monomer conversion and calculated polymerization shrinkage, mass and volume changes, apatite formation, PLS release, and the mechanical properties of novel resin composites with added calcium / strontium phosphate and polylysine.

### Monomer conversion and polymerization shrinkage

Sufficient monomer conversion is essential for reducing toxicity and enhancing mechanical properties of dental composites [23, 24]. It has been shown that the amount of toxic monomers eluted from such resin composites was substantially decreased when their monomer conversion was greater than 50% [25]. The monomer conversion of materials in this study were comparable with those of commercial products containing UDMA as the primary base monomer [26]. In addition, the conversion of Z250 obtained above is in agreement with that reported in an earlier study using a similar technique [27]. The higher conversion of experimental composites compared with that of Z250 is in agreement with a previous study [8]

Maximum final conversion of a composite is largely governed by the glass transition temperature (*T*_g_) of the monomers. As the monomers polymerize the T_g_ increases. Rate of conversion decreases dramatically when the *T*_g_ of the polymerizing mixture becomes coincident with the surrounding temperature [28]. High conversion is therefore generally observed in polymers consisting of flexible, low *T*_g_ monomers. The monomer *T*_g_ and the final room temperature conversions of homopolymers for Bis-GMA, UDMA, and TEGDMA are -8, -35 and -83°C and 39, 67 and 76% respectively [29]. Therefore, the final conversion values of Z250 (consisting of Bis-GMA as a primary monomer) and the experimental composites observed in the current study are within the expected range of these values.

Monomer conversion in this study was recorded at 1 mm depth, but in a clinical situation the cavity depth can be up to 4 mm. In a previous study, it was shown that the monomer conversion of Z250 was significantly below 50% if its thickness was greater than 1 mm [8]. This commercial material must therefore be placed in multiple layers complicating the clinical procedure. The same study also revealed that the conversion of the UDMA composites could be more than 50% up to 4 mm depth. This result, together with the very rapid cure, could facilitate the placement of the experimental composites.

The monomer conversion of the experimental composites was decreased upon the addition of PO. A possible explanation for this might be that this filler enhanced light scattering in the composites. The refractive index of the UDMA/TEGDMA mixture, glass, TSrP, MCPM, and PLS were approximately 1.47, 1.46, 1.65, 1.54, and 1.42 respectively [30–33]. The refractive indices mismatch between the monomer and particles will enhance scattering, thereby decreasing the amount of monomer conversion [34]. This then subsequently reduced calculated polymerization shrinkage in formulation with PO addition (F1, F2).

The calculated shrinkage of the experimental composites fell within the shrinkage observed with current commercial dental composites (1–6%) [35] and was only slightly greater than that of 2–2.4% observed for Z250 [8, 36]. The values were also in agreement with both calculated and experimental results obtained previously for similar formulations [20]. The calculation of polymerization shrinkage assumes that the shrinkage per mole of the C = C groups is constant throughout the polymerization process. Previous studies have shown reasonable agreement between calculated and experimental shrinkage results but that at lower and higher conversions, the calculation could slightly under [20] and over [8] estimate shrinkage.

### Mass and volume changes

Polymer-based dental restorations are continuously exposed to oral fluids. As a result, water sorption leads to changes in mechanical and physical properties. Water sorption is however required to enable components release to promote remineralizing or antibacterial effects [37, 38]. Water sorption is generally a diffusion-controlled process. Hence, the results in this study were plotted versus the square root of time. A previous study of calcium phosphate and CHX containing composites, demonstrated that the rate of water sorption was governed primarily by the hydrophilicity of the polymer phase, whereas the final water content was mainly determined by the amount of hydrophilic calcium phosphate [39].

Hydrophilicity of both PLS and MCPM probably led to an increase in early water sorption and, therefore, mass increase. This water would be expected to subsequently dissolve these components enabling their diffusion-controlled release and / or, in the case of MCPM, disproportionation into dicalcium phosphate and phosphoric acid. The dicalcium phosphate may then precipitate as lower solubility brushite that binds the water within the composite. The phosphoric acid may then diffuse to the tristrontium phosphate particles and react to form distrontium phosphate. Alternatively, it may be released into the aqueous surroundings.

In the current study, the mass changes observed with F3 and F4 were comparable with those observed for commercial materials [40]. Unlike F3 and F4, the mass changes of composites with PO (F1 and F2) were markedly affected by type of storage solution. The final negative mass changes of F1 and F2 composites in water might have been a consequence of greater mass loss compared with mass gain arising from water sorption. The buffer, in SBF would neutralize any released acid due to the reaction of MCPM with water. The phosphate counterions, however, may have supersaturated the solution and re-precipitate with calcium as apatite on the surface of the composites thereby reducing mass loss. The greater difference in mass in water versus SBF of F1 compared with F2 suggests the PLS is able to enhance the precipitation.

The volume of the experimental composites might have increased through the water expanding the polymerized resin matrix [39]. Although PLS addition enhanced mass change of the composites, it had minimal effect on volume change. Water sorption had therefore increased material density. Conversely, PO addition caused a large increase in volume. Previous studies suggest this may be due to the lower density brushite formation in the bulk of the materials forcing expansion of the surrounding polymer matrix [22, 39]. The final composite volume changes in water were comparable with polymerization shrinkage. In addition, the 1% difference in volume change observed at late time with F1 and F2 in water versus in SBF would be consistent with a 10 μm layer of apatite. This would be expected to help remineralization of >10 μm depth of surrounding acid affected dentin.

Water sorption may therefore promote component release and induce expansion to compensate polymerization shrinkage and stress [41]. It could, however, also negatively affect the long-term color stability and mechanical properties. Assessing these properties is therefore needed in future work.

### Apatite formation

A composite resin that promotes precipitation of apatite from ion-containing solutions may help seal gaps at the tooth-restoration interface and remineralize any residual demineralized dentin. The ion content of SBF is similar to that of dentinal fluid. Furthermore, BS ISO 23317: 2012 is a commonly used standard for assessment of the ability of materials to promote apatite precipitation from SBF [42, 43]. It was therefore used in the above study.

The formulations with MCPM and TSrP (F1 and F2) formed a visible apatite layer within 24 hr as was previously observed with the composites containing MCPM, TCP and CHX [7]. Hence, replacement of TCP and CHX with TSrP and PLS in this new study has not removed the apatite forming ability of the composites. An advantage of strontium over calcium phosphate would be increased radiopacity whilst the benefit of PLS instead of CHX would be enhanced eukaryotic cell compatibility [44, 45].

The apatite precipitation rate was faster with F1 than F2. This may be due to the effect of positively charged amine from PLS. This positively charged lysine group and extra water sorption may have encouraged the mineral release and attract the negatively charged pro-nucleation cluster, thus increasing the nucleation sites and the precipitation of the apatite [46]. This would be expected to enable remineralization of demineralized dentin and minimize the gap formation at the tooth-restoration interface. Typical clinical marginal gaps are ~10 μm [47] which is comparable to the thicknesses of the apatite layer. The rapid formation of apatite would be expected to enable immediate remineralizing of any residual demineralized dentin and help to minimize gap formation at the tooth-restoration interface.

### Polylysine release

Chlorhexidine (CHX) is a commonly used antibacterial agent for inhibition of dental biofilm formation. Recent studies, however, have demonstrated increasing antibiotic resistance to chlorhexidine [48, 49] and some severe hypersensitivity reactions [50, 51]. Polylysine is a FDA approved preservative that may potentially be used as alternative to address these issues. Previous studies have shown that to gain high release of CHX from composites requires addition of PO or hydrophilic monomer [8, 17]. The results of this study showed that the release of PLS was high regardless of PO addition. A possible explanation for this might be that PLS, compared with CHX, is a highly water-soluble compound that in itself can encourage high water sorption. In addition, PLS is a polyelectrolyte [52]. Therefore, its polymer chain will become positively charged upon dissolution in aqueous solutions. This creates a repulsive force between the repeating units in the polymer chain leading to polymer chain extension. This process may enable movement of the chain out of the composites into the surrounding water. The above data has demonstrated that the release of polylysine can be given by a slightly modified Fickian equation [53].

$$\Delta PLS=\Delta {PLS}_{0}+\Delta {PLS}_{\infty }\sqrt{\frac{2Dt}{\pi l^{2}}}$$ (10)

Where Δ*PLS* represents the change in cumulative PLS in the solution; Δ*PLS*_0_, early burst release; Δ*PLS*_∞_, maximum change in the solution; *D*, PLS diffusion coefficient; *t*, time; *l*, sample thickness. The early burst release may be due to the dissolution of PLS from the composite resin surface. This burst release may be expected to remove the residual bacteria and prevent the early rapid and spontaneous adhesion of bacteria. PLS may also subsequently be trapped and accumulate at the tooth restoration interface. This might prevent long term bacterial colonization.

### Biaxial flexural strength and modulus of elasticity

Successful dental composite restoration depends on sufficient mechanical strength to withstand the masticatory forces. According to BS ISO 4049, flexural strength from three-point bending test of 80 MPa is required for the restorative type dental composite [54]. The biaxial flexural strength obtained in the above study yields similar results to the three-point bending test but can be more reproducible [55]. The result from the above study therefore suggests the materials would pass the standard. Furthermore, the strength of the composites in the above studies was comparable or higher than that of calcium phosphate containing composites from previous publications [56, 57]. Addition of PO and PLS negatively affected mechanical properties of the composites as was expected. This could be due to the decrease in monomer conversion, the increase in water sorption, and the lack of adequate bond between these additives and the matrix [58]. Fillers treated with silane such as methacryloxypropyltrimethoxysilane can form a covalent bond, which is one of major factors that contributes to the strength of methacrylate-based composites [59]. PO and PLS fillers in this study were, however, intentionally not silane treated to enable their reaction and release. Although 4-META may provide a chemical bond between these fillers and matrix, this bond may be not sufficient to provide significant benefit. The releasing of components may also affect the long-term mechanical properties of the composites. With the provided level of PO, a previous study suggests, however, that the reduction in strength may be no more than that observed with Z250 [8]. This could be due to the formation of high-density dicalcium phosphates in the pores left after the releasing of reactive fillers [39].

## Conclusion

The addition of MCPM with TSrP and polylysine promoted hygroscopic expansion, apatite formation, and early polylysine release. These properties are expected to compensate the polymerization shrinkage, reseal any restoration gaps, and may help remineralize or kill residual demineralized dentin or bacteria in a cavity. Although addition of these fillers decreased the monomer conversion and strength of the composites, these properties were still within an acceptable range.

## Supporting Information

S1 File Raw data. Experimental composite raw data.
(XLSX)
